# Anti-tumour and anti-metastatic activity of 3-(P-Chlorophenyl)-2,3-Dihydro-3-Hydroxythiazolo (3,2-A)-Benzimidazole-2-acetic acid (WY-13,876).

**DOI:** 10.1038/bjc.1976.47

**Published:** 1976-03

**Authors:** R. L. Fenichel, F. J. Gregory, H. E. Alburn

## Abstract

Extensive investigation of 3-(p-chlorophenyl)-2,3-dihydro-3-hydroxythiazolo(3,2-alpha)-benzimidazole-2-acetic acid (Wy-13,876) in BDF1 mice implanted with Lewis lung tumour has shown that it is an effective anti-tumour and anti-metastatic agent. In vitro examination using HEp-2 human epidermal tumour cells has indicated that Wy-13,876 is not cytotoxic. When mice implanted with Lewis lung tumour and treated with Wy-13,876 are also injected with anti-thymocyte serum, an increase in lung metastases is observed suggesting that thymocyte activity is involved in the drug's mechanism of action. An increase in peripheral T lymphocytes observed in rats 18 h after a single oral dose of Wy-13,876 further supports this possibility. When Wy-13,876 is given to tumour -bearing mice in combination with low, ineffective doses of 5-fluorouracil or cyclophosphamide, further reduction of primary tumour growth is observed.


*Br. J. Cancer* (1976), **33**, 329

### ANTI-TUMOUR AND ANTI-METASTATIC ACTIVITY OF 3-(P-CHLOROPHENYL)-2,3-DIHYDRO-3-HYDROXYTHIAZOLO[3,2-*a*]-BENZIMIDAZOLE-2-ACETIC ACID (Wy-13,876)*

R. L. FENICHEL, F. J. GREGORY AND H. E. ALBURN

*From the Research Division, Wyeth Laboratories, Philadelphia, Pennsylvania* 19101, *U.S.A.*

Received 8 January 1975 Accepted 28 November 1975

**Summary.—Extensive investigation of 3-(p-chlorophenyl)-2,3-dihydro-3-hydroxythiazolo[3,2-*a*]-benzimidazole-2-acetic acid (Wy-13,876) in $BDF_1$ mice implanted with Lewis lung tumour has shown that it is an effective anti-tumour and anti-metastatic agent. *In vitro* examination using HEp-2 human epidermal tumour cells has indicated that Wy-13,876 is not cytotoxic. When mice implanted with Lewis lung tumour and treated with Wy-13,876 are also injected with anti-thymocyte serum, an increase in lung metastases is observed suggesting that thymocyte activity is involved in the drug's mechanism of action. An increase in peripheral T lymphocytes observed in rats 18 h after a single oral dose of Wy-13,876 further supports this possibility. When Wy-13,876 is given to tumour-bearing mice in combination with low, ineffective doses of 5-fluorouracil or cyclophosphamide, further reduction of primary tumour growth is observed.**

IT HAS BEEN reported (Stjernswärd *et al.*, 1972) that radiation for mammary carcinoma lowers the circulating level of thymus (T) lymphocytes for at least 1 year, and these workers suggest that the observed increase in metastases might be related to the lymphopaenia. Other studies (Renoux and Renoux, 1972) have indicated that levamisole inhibits the growth and subsequent lung metastases of Lewis lung (3LL) tumour in C57Bl mice. Using the Lewis lung (3LL) tumour in $BDF_1$ mice, we have modified a model system described by James and Salsbury (1974) to study the anti-tumour and anti-metastatic activity of new compounds including potential anti-helminthic agents chemically related to levamisole (Bell and Wei, 1976). The most effective of these was found to be 3-(p-chlorophenyl)-2,3-dihydro-3-hydroxythiazolo [3,2-*a*]-benzimidazole-2-acetic acid (Wy-13,876, NSC 208828).

Wy-13,876

### MATERIALS AND METHODS

Six to 8-week-old male $BDF_1$ mice weighing 19–21 g were used for the tumour studies. This strain is used to conform with Protocol 1.400 of the Drug Research and Development Division of Cancer Treatment, National Cancer Institute. Growth of the Lewis lung carcinoma in the $F_1$ generation of this hybrid is completely comparable with that obtained in the syngeneic C57Bl mouse. The latter line was always employed for stock tumour growth (Geran *et al.*, 1972). Subcutaneous trochar implants of 2–4 mm fragments of 3LL tumour were made in the axillary

* A portion of this study was presented at the 66th Annual Meeting of the American Association for Cancer Research, 7–11 May, 1975, San Diego, California.

region. Starting 24 h later the mice received daily intraperitoneal injections of Wy-13,876, or levamisole (kindly supplied by Janssen Laboratories, Belgium), suspended in a solution composed of 1 part of 0·5% carboxymethylcellulose (CMC, Methocel, Dow Chemical Company, Midland, Mich.) and 2 parts of physiological saline, while the control animals received only the CMC–physiological saline solution. At the end of the experimental period the mice were killed, the tumour and lungs excised, the primary tumours weighed for the determination of T/C ratios (mean tumour weight for treated divided by mean tumour weight for control animals × 100) and the lungs examined for metastases, either by observation of the total lung surface with a dissecting microscope or by histopathological examination. The histopathological examination involved making serial transverse sections of the lungs at intervals of several millimetres until the entire lung mass was used. Twelve to 14 sections were obtained per lung. A haematoxylin and eosin stain was used and the slides were examined microscopically at both low and high magnification so as to identify all metastases, including early ones. All slides were coded before examination by the pathologist to create a blind study.

In order to test the importance of viable circulating lymphocytes for the antitumour activity of Wy-13,876 and levamisole, antithymocyte serum (ATS, Microbiological Associates, Rockville, Md) was administered undiluted by the intraperitoneal route to separate groups of mice implanted with Lewis lung (3LL) tumour at doses of 0·1 ml/mouse one day before tumour implantation and 1 and 5 days after implantation for the first week then twice a week for the remainder of the experiment. Positive control groups that were given cyclophosphamide (Cytoxan, Mead Johnson Laboratories, Evansville, Ind.) as well as control groups that did not receive any compound, with and without the injection of ATS, were also included in this experiment. Treatment was stopped on Day 14 and the animals were kept for 4 additional days before killing. The primary tumours were weighed and the lungs were preserved in formalin and subjected to examination under a dissecting microscope for the determination of the number of lung metastases.

To study the effect of Wy-13,876 or levamisole on T lymphocyte formation, male CD® rats in groups of 4 were given various doses of the drugs suspended in water by gavage, the control rats receiving only water. The rats were fasted overnight, and 18 h after drug administration, blood samples were taken by cardiac puncture using heparinized vacutainers (Becton Dickinson and Co., Rutherford, N.J.). The blood from the rats in each dosage group was pooled, diluted with an equal volume of Hanks' modified balanced salt solution (HMBSS), 8 ml layered on a 4 ml gradient containing 10 parts of 50% Hypaque with 16 parts of cold 2% methylcellulose (15 ct/s Dow Chemical Co., Midland, Mich.) and the lymphocytes separated at the interface after centrifugation according to the procedure of Boyum (1968). The lymphocytes were then washed twice with HMBSS.

The incubation system for rosette formation, patterned after the procedure of Siegel and Sherman (1972), consists of 0·1 ml of lymphocyte suspension ($1{\cdot}6 \times 10^6$ cells/ml), 0·1 ml of guinea-pig red blood cells ($6{\cdot}4 \times 10^6$ cells/ml) and 0·1 ml of foetal calf serum in HMBSS. The tubes, set up in triplicate for control and experimental groups, are incubated in a shaker bath for 60 min at 37°C, the number of rosettes per 100 thymocytes from each of the 3 tubes counted under the microscope, and the results expressed as the mean value of rosettes (T lymphocytes) for the experimental group, divided by the mean value for the control group.

A series of experiments was also conducted with mice using the described Lewis lung system in which combination of Wy-13,876 with low anti-tumour doses of cyclophosphamide and 5-fluorouracil (Roche Laboratories, Nutley, N.J.) were used.

Cytotoxicity of Wy-13,876 was determined using HEp-2 human epidermal tumour grown as monolayers in tissue culture flasks in a basal serum medium according to the established procedure of Geran *et al.* (1972). Various concentrations of Wy-13,876 were prepared in demineralized water and added to the culture tubes, which were then incubated at 37°C until at least a six-fold increase in total protein was obtained in the control tubes, as indicated by the Lowry assay. Compounds are considered active in this test if they show an $ED_{50}$ of $< 4\ \mu g/ml$ as an average of 2 assays.

Daily intraperitoneal injections of different doses of Wy-13,876 were given to separate groups of 10 $BDF_1$ mice for 10 consecutive days to determine the *in vivo* toxicity of the compound according to the procedure of Reed and Muench (1938).

RESULTS

Wy-13,876 was not inhibitory to HEp-2 (human epidermal tumour) *in vitro* below 198 μg/ml, indicating that the compound is not cytotoxic. Tests to determine *in vivo* toxicity of Wy-13,876 (Table I)

TABLE I.—*Toxicity of Wy*-13,876 *in* $BDF_1$ *Mice*

| Dose (mg/kg) | No. of doses | Cumulative No. alive | Cumulative No. dead | % Dead |
|---|---|---|---|---|
| 400 | 10 | 3 | 18 | 85·7 |
| 300 | 10 | 6 | 11 | 64·7 |
| 200 | 10 | 12 | 4 | 25·0 |
| 100 | 10 | 22 | 0 | 0·0 |

revealed that it could be administered safely by the intraperitoneal route to mice at doses as high as 200 mg/kg/day for 10 days. The chronic $LD_{50}$ for Wy-13,876 with 10 daily doses was 263 mg/kg. The highest repeated dose of levamisole that could be given under our test conditions was 25 mg/kg/day.

The results of a comparative study of Wy-13,876 with a positive control cyclophosphamide and with levamisole (Table II) show some inhibition of primary tumour growth by Wy-13,876.

Table III shows the dose responses obtained for Wy-13,876 and levamisole. No significant inhibition was observed with Wy-13,876 at 12·5 or 25 mg/kg. At 50 and 100 mg/kg inhibition of primary tumour growth is seen and at 150 mg/kg a significant T/C ratio of 37% is observed. Levamisole did not depress primary tumour growth at either 12·5 or 25 mg/kg.

The effect of Wy-13,876 on primary tumour growth and lung metastases, as monitored by histopathological examination, is shown in Table IV. Primary tumour weight reduction was seen at both the 100 and 150 mg/kg doses, although only the 150 mg/kg dose brought the T/C value below the 42% level. The histopathological study of the lungs from these mice indicated that at the 150 mg/kg dose, 6 of 10 mice showed no metastases whereas the lungs of the remaining 4 mice showed 1, or 3 to 5 indications of small metastases. At the 100 mg/kg dose, 2 mice showed no metastases; 4 mice, 3–5 tiny metastases; 2 mice, 6–9 metastases and the final 2, 10–21 metastases. The cyclophosphamide control showed no lung metastases and the negative controls showed a spectrum of metastatic involvement. In comparison with the lungs from the CMC control mice 8% metastases were observed in the lungs of the mice dosed with 150 mg/kg of Wy-13,876 and 52% in the lungs of those given 100 mg/kg.

Table V shows the results of the experiment in which ATS was used to suppress thymocyte function in mice receiving either Wy-13,876, levamisole, cyclophosphamide or CMC control solu-

TABLE II.—*Activity of Wy*-13,876, *Levamisole and Cyclophosphamide against Lewis Lung (3LL) Tumour*

| Compound | Dose (mg/kg) | Mean wt chg. (g) | D/T* | N | % (T/C)† | Mean tumour wt (g) ± s.e.‡ | *P* value |
|---|---|---|---|---|---|---|---|
| Wy-13,876 | 100 | +1·8 | 1/10 | 9 | **34** | 0·471±0·2 | <0·05 |
| Levamisole | 25 | +0·9 | 1/10 | 9 | 79 | 1·089±0·2 | N.S. |
| Cyclophosphamide | 20 | 0·0 | 0/10 | 10 | **17** | 0·234±0·1 | <0·001 |
| Control§ | — | +2·0 | 0/20 | 20 | — | 1·380±0·2 | — |

* Dead/total number of animals.
† Mean tumour weight for treated/mean tumour weight for control mice × 100.
‡ Standard error.
§ Dosed with 0·2% CMC in physiological saline.
In bold type: T/C ratio ⩽42%, *i.e.* active dose of compound.

TABLE III.—*Dose Response of Wy-13,876 and Activity of Levamisole against Lewis Lung (3LL) Tumour*

| Compound | Dose (mg/kg) | Mean wt chg. (g) | D/T* | N | % (T/C)† | Mean tumour wt (g) ± s.e.‡ | *P* Value |
|---|---|---|---|---|---|---|---|
| Wy-13,876 | 12·5 | +1·6 | 0/10 | 10 | 66 | 1·68±0·2 | <0·01 |
| Wy-13,876 | 25 | +1·2 | 0/10 | 10 | 74 | 1·89±0·3 | <0·1 |
| Wy-13,876 | 50 | +1·2 | 0/10 | 10 | 48 | 1·23±0·1 | <0·001 |
| Wy-13,876 | 100 | +0·7 | 2/10 | 8 | 47 | 1·20±0·2 | <0·001 |
| Wy-13,876 | 150 | −0·5 | 2/10 | 8 | **37** | 0·95±0·1 | <0·001 |
| Wy-13,876 | 200 | +0·5 | 2/10 | 8 | 67 | 1·70±0·2 | <0·02 |
| Levamisole | 12·5 | +0·6 | 0/10 | 10 | 78 | 1·99±0·2 | <0·1 |
| Levamisole | 25 | +1·3 | 4/10 | 6 | 71 | 1·80±0·3 | <0·1 |
| Control§ | — | +2·2 | 0/20 | 20 | — | 2·54±0·2 | — |

* Dead/total number of animals.
† Mean tumour weight for treated/mean tumour weight for control mice × 100.
‡ Standard error.
§ Dosed with 0·2% CMC in physiological saline.
In bold type: T/C ratio ⩽ 42%, *i.e.* active dose of compound.

TABLE IV.—*Activity of Wy-13,876 against Lewis Lung (3LL) Tumour and Histopathological Evaluation of Lung Metastases*

| | | | | | | | | Mice with lung metastases | | | | | |
|---|---|---|---|---|---|---|---|---|---|---|---|---|---|
| | | | | | | | | No. of metastases | | | | | |
| Compound | Dose (mg/kg) | Mean wt chg. (g) | D/T* | N | % (T/C)† | Mean tumour wt (g) ± s.e.‡ | *P* Value | 0 | 1–2 | 3–5 | 6–9 | 10–21 | Metastases (% of cont.) |
| Wy-13,876 | 150 | −0·3 | 0/10 | 10 | **41** | 0·75±0·3 | <0·02 | 6 | 1 | 3 | 0 | 0 | 8 |
| Wy-13,876 | 100 | +0·3 | 0/10 | 10 | 54 | 1·01±0·2 | <0·05 | 2 | 0 | 4 | 2 | 2 | 52 |
| Cyclophosphamide | 20 | −2·0 | 0/10 | 10 | **17** | 0·32±0·1 | <0·001 | 10 | 0 | 0 | 0 | 0 | 0 |
| CMC control | — | −0·6 | 0/20 | 20 | — | 1·86±0·2 | — | 1 | 4 | 6 | 2 | 6 | 100 |

* Dead/total number of animals.
† Mean tumour weight for treated/mean tumour weight for control mice × 100.
‡ Standard error.
In bold type: T/C ratio ⩽ 42%, *i.e.* active dose of compound.

TABLE V.—*Effect of Anti-thymocyte Serum on Primary Tumour Growth and Lung Metastases in Lewis Lung (3LL) Tumour-implanted Mice Treated with Wy-13,876 or Levamisole*

| Compound | Dose (mg/kg) | ATS§ | Mean wt chg. (g) | D/T* | N | % (T/C)† | Mean tumour wt (g)±s.e.‡ | *P* Value | Average no. of lung metastases¶ |
|---|---|---|---|---|---|---|---|---|---|
| Wy-13,876 | 100 | No | −0·5 | 1/9 | 9 | 48 | 2·03±0·6 | <0·05 | 2·4 |
| Wy-13,876 | 100 | Yes | −0·6 | 4/10 | 6 | 66 | 1·98±0·5 | N.S. | 6·3 |
| Levamisole | 25 | No | −0·3 | 2/10 | 8 | 59 | 2·54±0·5 | <0·1 | 3·4 |
| Levamisole | 25 | Yes | −1·8 | 5/10 | 5 | 63 | 1·92±0·8 | N.S. | 2·5 |
| Cyclophosphamide | 20 | No | −3·1 | 0/9 | 9 | **23** | 1·00±0·2 | <0·001 | 0 |
| Cyclophosphamide | 20 | Yes | — | 9/10 | 1 | — | — | — | 6·0 |
| CMC control | — | No | −0·2 | 0/9** | 9 | — | 4·26±0·6 | — | 12·0 |
| CMC control | — | Yes | −0·8 | 2/10 | 8 | — | 3·02±0·4 | — | 17·5 |

* Dead/total number of animals.
† Mean tumour weight for treated/mean tumour weight for control mice × 100.
‡ Standard error.
§ Antithymocyte serum (Microbiological Associates).
¶ As observed with a dissecting microscope.
** One animal killed to evaluate metastatic spread on Day 14.
In bold type: T/C ratio ⩽ 42%, *i.e.* active dose of compound.

tions, in comparison with similarly treated mice that did not receive ATS. In this experiment the mice treated with Wy-13,876 did not show any difference in primary tumour weight, regardless of whether they received ATS. A difference in primary tumour weight is observed for those animals that received levamisole. A lesser number of lung metastases was found for the mice that received Wy-13,876, but not ATS, in comparison with those which received Wy-13,876 plus ATS. Little difference was observed between the levamisole-treated mice whether they did or did not receive ATS.

Lung metastases were not found in mice that received cyclophosphamide without ATS, but 9 of 10 of the cytoxan treated mice that also received ATS died. Increased lung metastases were also found in the ATS-treated control mice.

An initial examination of the effects of different doses of Wy-13,876 or levamisole on T lymphocytes (Table VI), as demonstrated by rosette formation, showed some activity for Wy-13,876 at 75 mg/kg, and a doubling of T lymphocytes in comparison with the controls at 100 and 200 mg/kg. Levamisole did not increase T lymphocytes at 75 mg/kg, but slight activity at 100 mg/kg and good activity at 150 mg/kg were observed.

Table VII shows the effects of combinations of Wy-13,876 or levamisole with 5-fluorouracil on primary tumour growth in mice. In this experiment a good T/C ratio was found for the 150 mg/kg dose of Wy-13,876, and its combination with a low dose of 5-fluorouracil (10 mg/kg) substantially lowered the T/C ratio.

Analysis of the tumour weight difference between the Wy-13,876 treated group and the group treated with Wy-13,876 and 5-fluorouracil did not show a significant statistical difference between the 2 groups. A comparatively low T/C

TABLE VI.—*Effect of Wy*-13,876 *and Levamisole on T-Lymphocytes as Measured by Rosette Formation* 18 *h after a Single Oral Dose*

| Compound | Dose (mg/kg) | No. of adhering thymocytes (Mean ± S.D. of 3 tubes) | Rosette formation* E/C ratio |
|---|---|---|---|
| Wy-13,876 | 75 | 4·3±0·6 | 1·4 |
| Levamisole | 75 | 2·3±0·6 | 0·8 |
| Control | — | 3·0±1·0 | — |
| Wy-13,876 | 100 | 7·0±2·0 | 1·9 |
| Levamisole | 100 | 5·3±0·6 | 1·4 |
| Control | — | 3·7±0·6 | — |
| Wy-13,876 | 200 | 6·3±1·1 | 2·1 |
| Levamisole | 150 | 5·0±0 | 1·7 |
| Control | — | 3·0±0 | — |

* Number of rosettes per 100 thymocytes are determined for 3 groups of 100 cells from 3 separate incubation tubes for the experimental and control groups and the means of these values are used to calculate the ratio.

TABLE VII.—*Evaluation of the Capacity of Low Doses of* 5-*Fluorouracil to Potentiate the Anti-Lewis Lung* (3*LL*) *Tumour Activity of Wy*-13,876 *and Levamisole*

| Compound | Dose (mg/kg) | Mean wt chg. (g) | D/T* | N | % (T/C)† | Mean tumour wt (g) ± s.e.‡ | *P* Value |
|---|---|---|---|---|---|---|---|
| Wy-13,876 | 150 | +0·2 | 0/10 | 10 | **39** | 0·60±0·1 | <0·001 |
| Levamisole | 25 | −0·1 | 1/10 | 9 | **35** | 0·54±0·2 | <0·01 |
| 5-Fluorouracil | 10 | −4·4 | 0/10 | 10 | 53 | 0·80±0·2 | <0·02 |
| Wy-13,876 + 5-Fluorouracil | 150<br>10 | −3·2 | 0/10 | 10 | **25** | 0·37±0·1 | <0·01 |
| Levamisole + 5-Fluorouracil | 25<br>10 | −4·4 | 1/10 | 10 | **36** | 0·55±0·1 | <0·001 |
| CMC control | — | −0·5 | 1/20 | 18§ | — | 1·52±0·2 | — |

* Dead/total number of animals.
† Mean tumour weight in treated/mean tumour weight for control mice × 100.
‡ Standard error.
§ One " no take " eliminated from calculation.
In bold type: T/C ratio ⩽ 42%, *i.e.* active dose of compound.

TABLE VIII.—*Evaluation of the Capacity of Low Doses of Cyclophosphamide to Potentiate the Anti-Lewis Lung (3LL) Tumour Activity of Wy-*13,876

| Compound | Dose (mg/kg) | Mean wt chg. (g) | D/T* | N | % (T/C)† | Mean tumour wt (g) ± s.e.‡ | *P* Value |
|---|---|---|---|---|---|---|---|
| Wy-13,876 | 100 | 0·0 | 0/10 | 10 | 53 | 0·98±0·2 | <0·01 |
| Cyclophosphamide | 10 | +0·1 | 0/10 | 10 | 90 | 1·66±0·3 | N.S. |
| Cyclophosphamide | 5 | −0·6 | 0/10 | 10 | 75 | 1·38±0·3 | N.S. |
| Wy-13,876 + Cyclophosphamide | 100<br>10 | −1·0 | 0/9 | 9 | **38** | 0·70±0·1 | <0·001 |
| Wy-13,876 + Cyclophosphamide | 100<br>5 | −1·6 | 0/10 | 10 | 44 | 0·82±0·2 | <0·01 |
| CMC control | — | −0·2 | 0/20 | 20 | — | 1·84±0·2 | — |

* Dead/total number of animals.
† Mean tumour weight in treated/mean tumour weight for control mice × 100.
‡ Standard error.
In bold type: T/C ratio ⩽ 42% *i.e.* active dose of compound.

value was obtained in the experiment in which levamisole was given to tumour-implanted mice; a very small reduction in T/C ratio was found when levamisole was combined with the low dose of 5-fluorouracil.

The results with 2 low doses of cyclophosphamide, 5 and 10 mg/kg, in combination with a 100 mg/kg dose of Wy-13,876 indicated a reduction of primary tumour growth at both cyclophosphamide levels (Table VIII), but the difference between these 2 groups and the group that received Wy-13,876 alone was not statistically significant.

In no instance in these experiments did the weight changes observed in mice treated with Wy-13,876 exceed those observed for the controls.

### DISCUSSION

The results show that Wy-13,876 inhibits primary Lewis lung tumour growth in mice and has significant activity in preventing lung metastases. Parallel studies indicate that levamisole has only very moderate activity in preventing primary tumour growth under these conditions. The dose response examination of the activity of Wy-13,876 shows optimal anti-tumour activity at 150 mg/kg, and a good indication of activity is observed at the 50 and 100 mg/kg doses.

In these experiments tumour weight reductions were frequently observed which were statistically significant but were insufficient to meet the National Cancer Institute protocol requirement for activity, that is T/C ratios of less than 42%. In the Tables of results where the data did meet the NCI criterion the T/C values are underlined.

Wy-13,876 did not show cytotoxic activity against HEp-2 human epidermal tumour cells (Geran *et al.*, 1972) and the chronic $LD_{50}$ for this compound in mice of 263 mg/kg suggests a good margin of safety for its use.

Wy-13,876 treated mice injected with ATS did not show any significant difference in primary tumour weight but the increase in lung metastases suggests that cell-mediated immune mechanisms, possibly through increased activity of T lymphocytes, plays a part in the reduction of these metastases.

The average number of lung metastases found for the levamisole + ATS-treated mice was slightly less than the number found for the matched levamisole-treated animals. These results can be explained either by the loss of 5 of 10 mice in the ATS-treated group, or by the possibility that levamisole is a weak immunostimulant and thymocytes play only a minimal role in its mechanism of action. The first reason seems to be the more likely one since the differential in the average number of metastases in the control ATS-treated and untreated group of mice is much greater and the number of lung metastases in the control groups is very much higher. The rosette test, which indicates the capacity to stimulate the generation of T lymphocytes, shows that both Wy-13,876 and levamisole increase T lymphocyte levels in rats. This experiment further suggests that increased T lymphocyte levels may be involved in the anti-metastatic mechanism.

The final experiments concern a study of the capacity of Wy-13,876 to potentiate the anti-tumour activity of 5-fluorouracil and cyclophosphamide and the effect of levamisole on 5-fluorouracil activity. Chirigos, Pearson and Pryor (1973) have reported on the value of combining chemotherapy and immunostimulation in a murine leukaemia and they have shown that, when levamisole is combined with low doses of 1,3 bis(2-chloroethyl)-1-nitrosourea, a higher percentage of long-term survivors is found. In our experiments a low dose of 5-fluorouracil had at least an additive anti-tumour effect with Wy-13,876, as indicated by the T/C ratios, but it did not affect the activity of levamisole. Low doses of cyclophosphamide also appeared to augment the anti-tumour activity of Wy-13,876 by this criterion.

We thank Dr Peter Wei for the synthesis of Wy-13,876, Dr Walter E. Tucker for developing the histopathological technique for the examination of lung tissue, Dr Allen J. Steinberg (Philadelphia General Hospital) for the evaluation of the lung tissues reported in this study and Manuela Selles and Richard L. Bloom for valuable technical assistance. Special acknowledgment is made to Shirley Fenichel, whose indication of the importance of a resistance factor in cancer, and whose encouragement, made this work possible.

### REFERENCES

Bell, S. J. & Wei, P. (1976) Syntheses of Heterocyclic Fused Thiazole Acetic Acids. II. *J. med. Chem.* In the press.

Boyum, A. (1968) Isolation of Leucocytes from Human Blood Further Observations. *Scand. J. clin. Lab. Invest. Suppl.* 97, **21**, 31.

Chirigos, M. A., Pearson, J. W. & Pryor, J. (1973) Augmentation of Chemotherapeutically Induced Remission of a Murine Leukemia by a Chemical Immunoadjuvant. *Cancer Res.*, **33**, 2615.

Geran, R. I., Greenberg, N. H., Macdonald, M. M., Schumacher, A. M. & Abbott, B. J. (1972) Protocols for Screening Chemical Agents and Natural Products Against Animal Tumors and Other Biological Systems. *Cancer chemother. Rep.*, **3**, 1.

James, S. E. & Salsbury, A. J. (1974) Effect of (±)-1,2-Bis(3,5-dioxopiperazin-1-yl)propane on Tumor Blood Vessels and Its Relationship to the Antimetastatic Effect in the Lewis Lung Carcinoma. *Cancer Res.*, **34**, 839.

Reed, I. J. & Muench, H. (1938) A Simple Method of Estimating Fifty Per Cent Endpoints. *Am. J. Hyg.*, **27**, 493.

Renoux, G. & Renoux, M. (1972) Levamisole Inhibits and Cures a Solid Malignant Tumor and its Pulmonary Metastases in Mice. *Nature, New Biol.*, **240**, 217.

Siegel, I. & Sherman, W. B. (1972) The Interaction of Lymphocytes with Autologous Red Cells. *J. Allergy clin. Immunol.*, **50**, 65.

Stjernswärd, J., Jondal, M., Vansky, F. & Sealy, R. (1972) Lymphopenia and Change in Distribution of Human B and T Lymphocytes in Peripheral Blood Induced by Irradiation for Mammary Carcinoma. *Lancet*, ii, 1352.


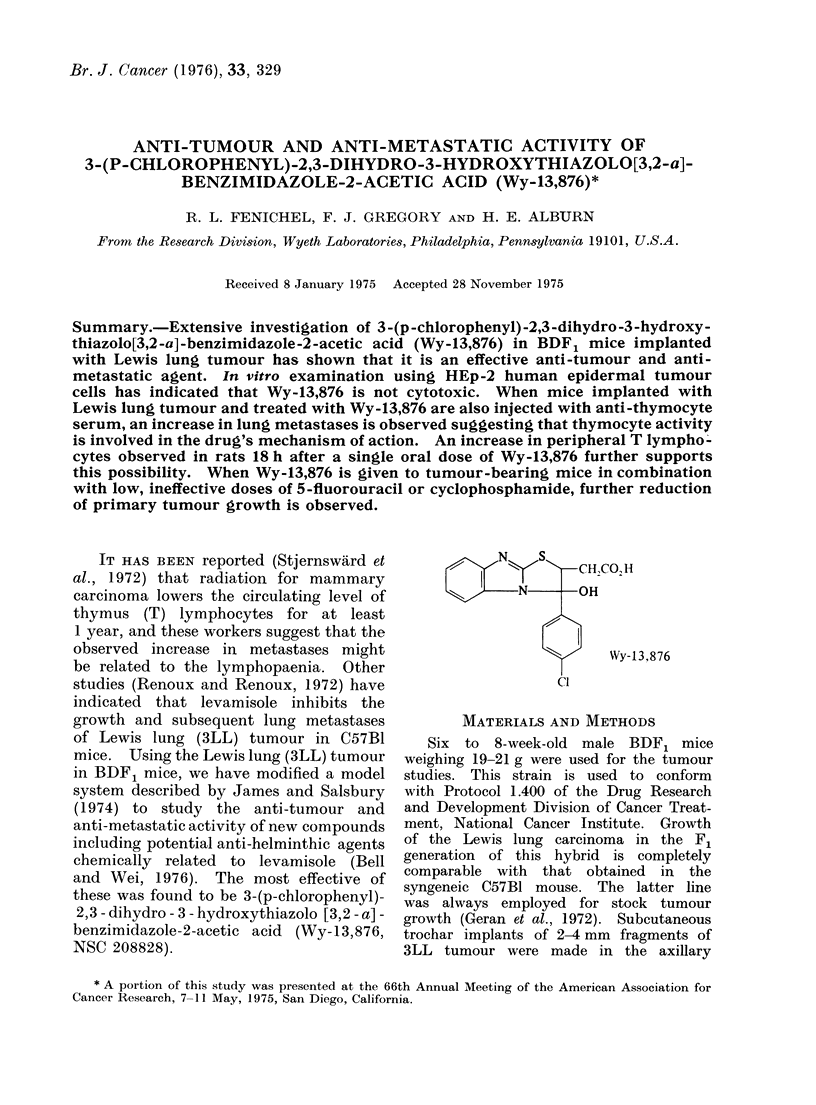

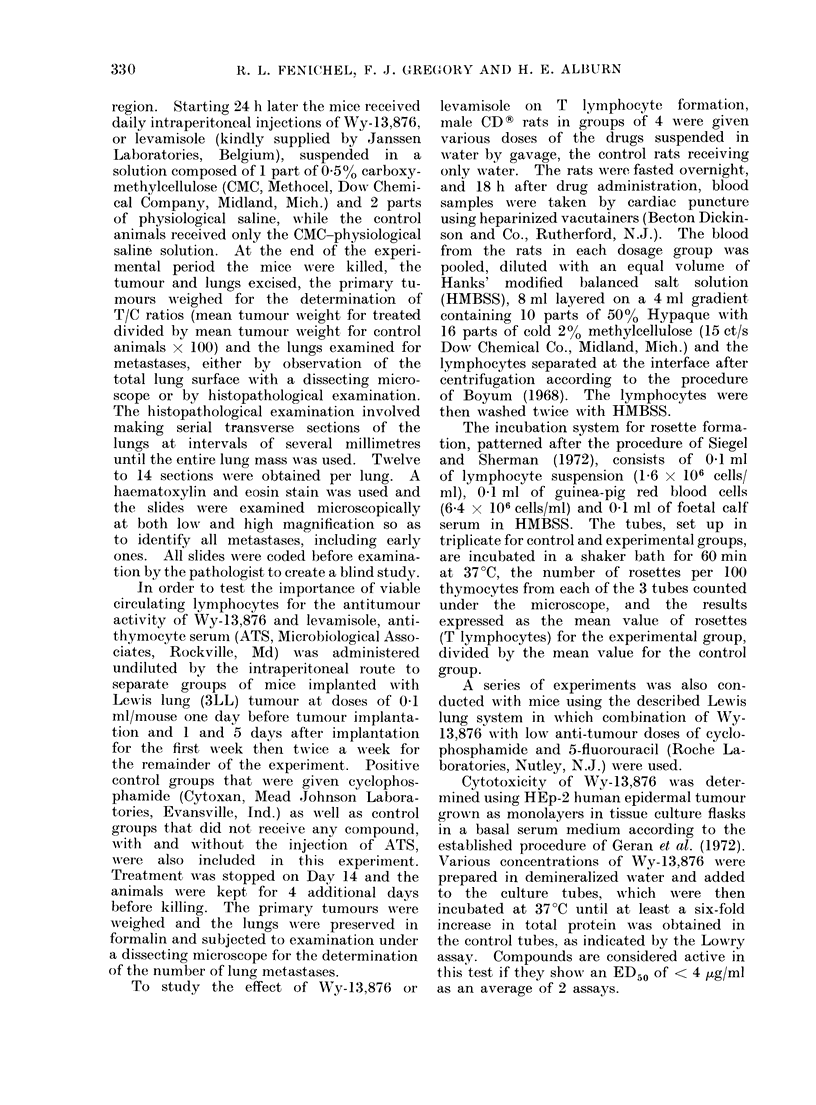

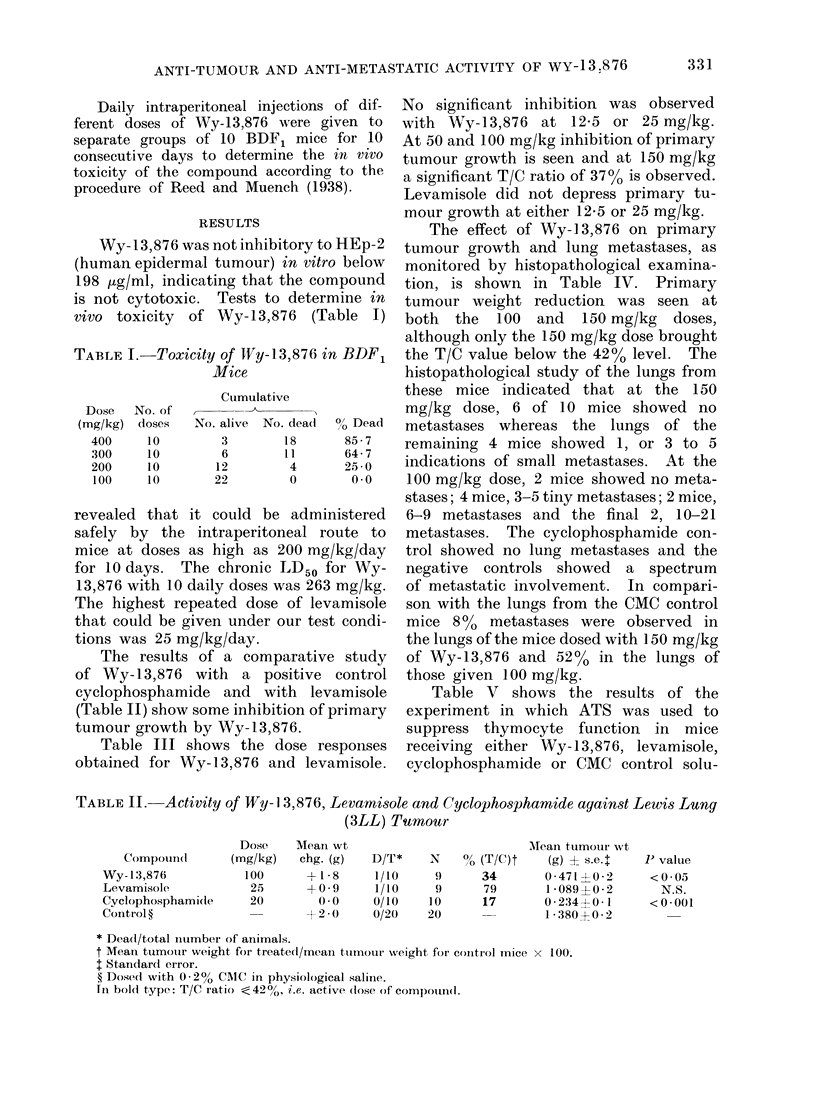

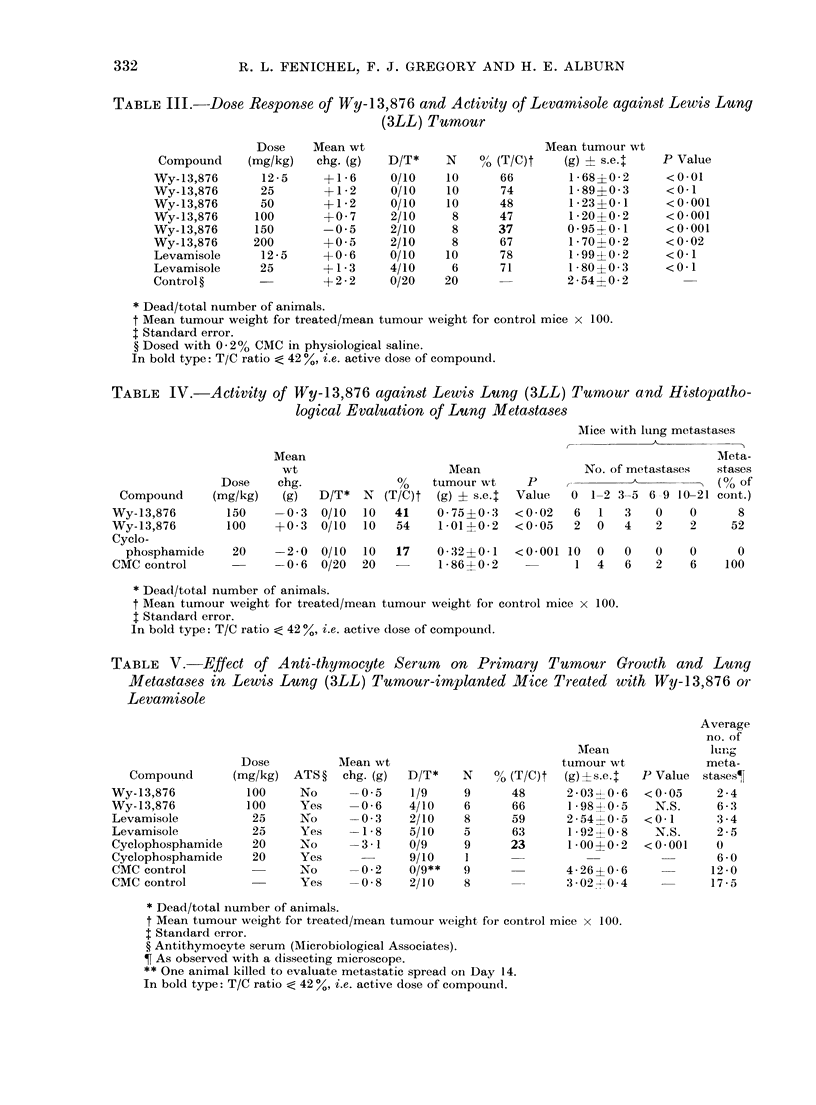

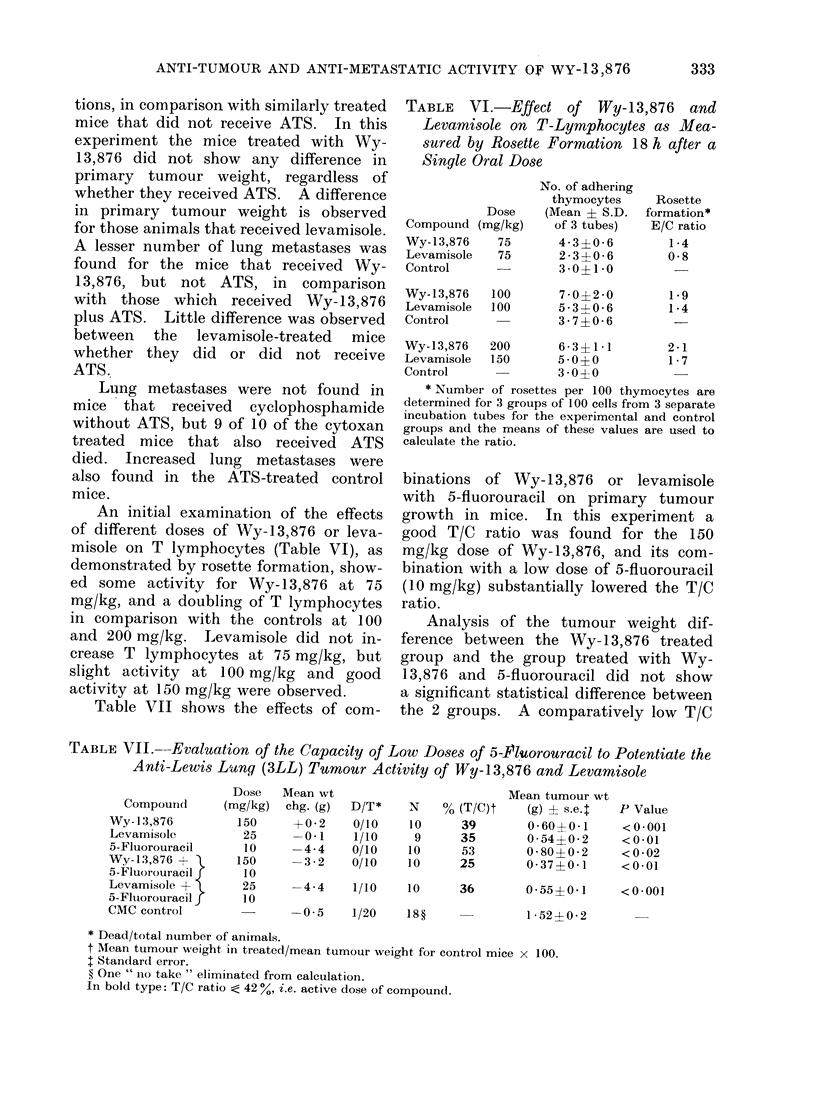

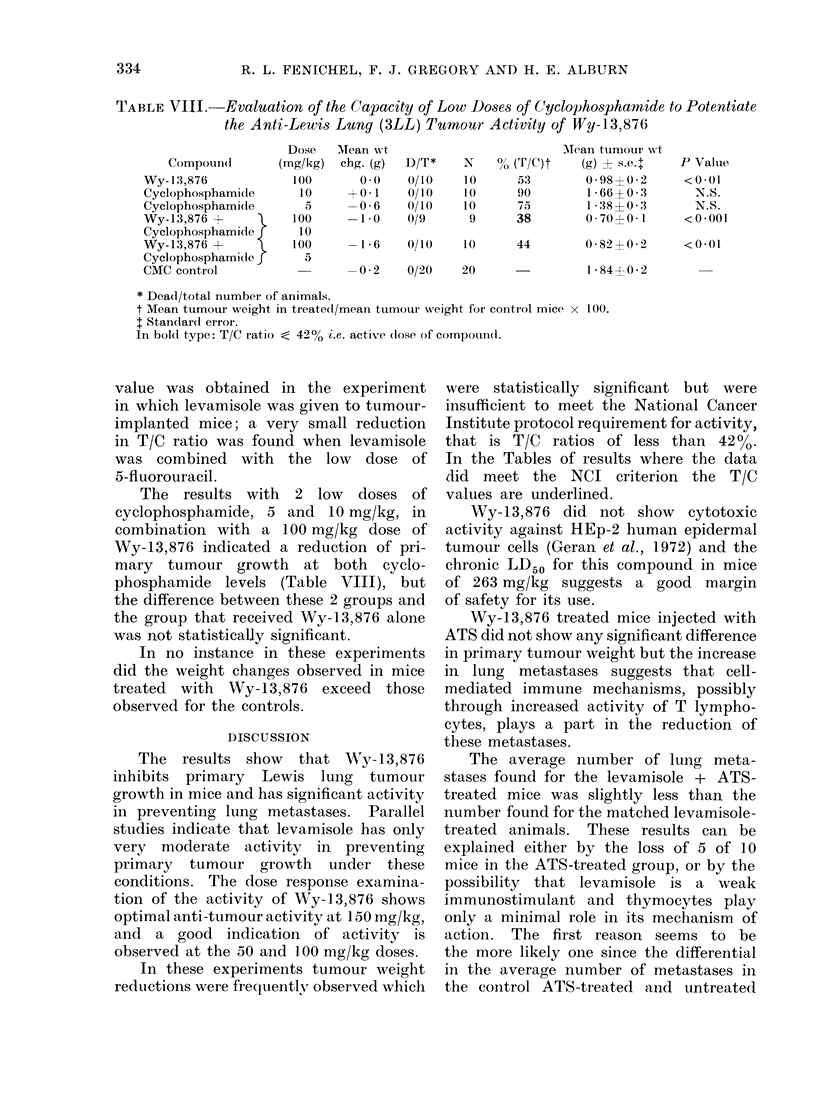

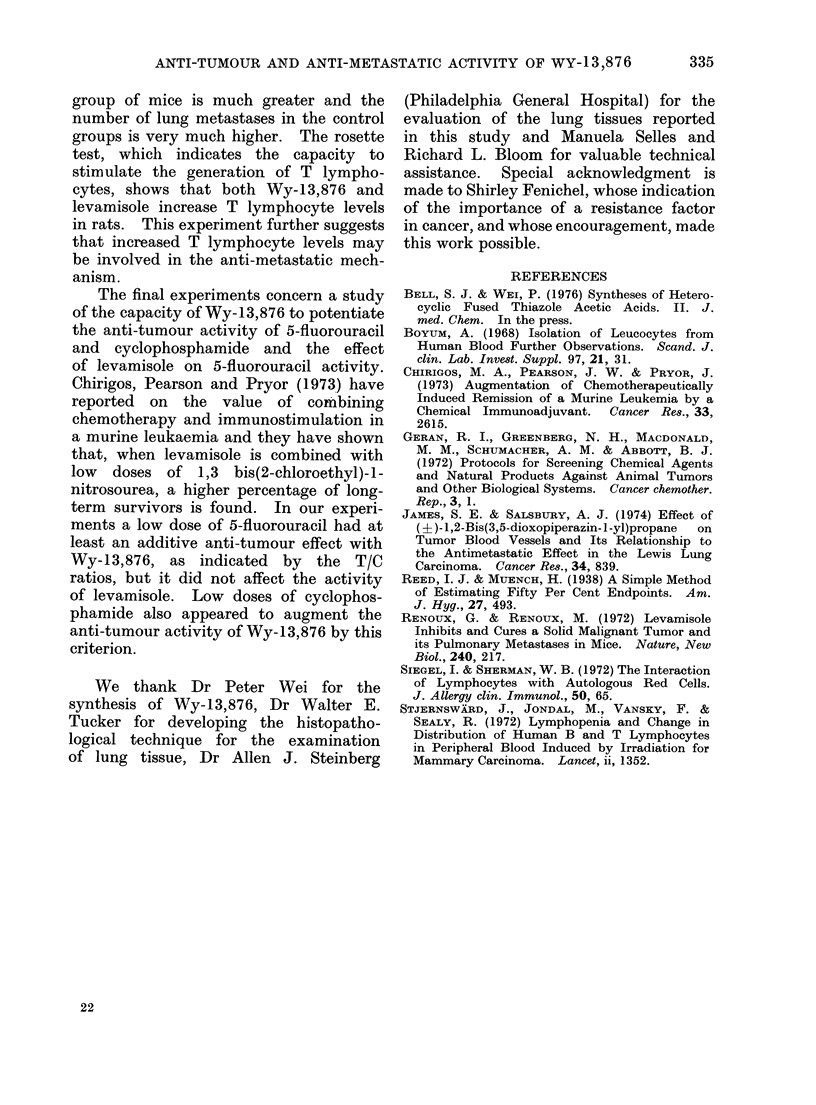


## References

[OCR_00928] Chirigos M. A., Pearson J. W., Pryor J. (1973). Augmentation of chemotherapeutically induced remission of a murine leukemia by a chemical immunoadjuvant.. Cancer Res.

[OCR_00943] James S. E., Salsbury A. J. (1974). Effect of (plus or minus)-1,2-bis(3,5-dioxopiperazin-1-yl)propane on tumor blood vessels and its relationship to the antimetastatic effect in the Lewis lung carcinoma.. Cancer Res.

[OCR_00955] Renoux G., Renoux M. (1972). Levamisole inhibits and cures a solid malignant tumour and its pulmonary metastases in mice.. Nat New Biol.

[OCR_00961] Siegel I., Sherman W. B. (1972). The interaction of lymphocytes with autologous red cells.. J Allergy Clin Immunol.

[OCR_00966] Stjernswärd J., Jondal M., Vánky F., Wigzell H., Sealy R. (1972). Lymphopenia and change in distribution of human B and T lymphocytes in peripheral blood induced by irradiation for mammary carcinoma.. Lancet.

